# Clinical Outcomes and Predictive Factors in Adrenalectomy: A Retrospective Cohort Study

**DOI:** 10.30699/ijp.2025.2052015.3407

**Published:** 2025-11-11

**Authors:** Seyed Alireza Mirsharifi, Shirzad Nasiri, Seyed Mohammad Tavangar, Mohammadamin Parsaei

**Affiliations:** 1 Department of General Surgery, School of Medicine, Dr. Shariati Hospital, Tehran University of Medical Sciences, Tehran, Iran; 2 Department of Pathology, Dr. Shariati Hospital, Tehran University of Medical Sciences, Tehran, Iran; 3 Breastfeeding Research Center, Family Health Research Institute, Tehran University of Medical Sciences, Tehran, Iran

**Keywords:** Adrenal Tumor, Adrenalectomy, Aldosteronism, Cushing, Hypertension, Pheochromocytoma

## Abstract

**Background & Objective:**

Adrenal tumors present a notable prevalence of 4-7% in individuals above 40 years old. Current guidelines recommend adrenalectomy for hormone-secreting, potentially malignant, or large (>4 cm) lesions. However, the outcomes of adrenalectomy and their clinical-pathological associations remain poorly defined. This study assessed adrenalectomy outcomes and explored its clinical, demographic, and pathologic correlates.

**Methods:**

In this retrospective cohort study, the medical records of all patients who underwent adrenalectomy from March 2016 to March 2021 at a referral center in Tehran were reviewed. Also, a clinical follow-up via telephone was conducted. The chi-square test, independent t-test, and analysis of variance were utilized for statistical analysis.

**Results:**

Data from a total of 75 patients (55 females) were reviewed. The mean age of the participants was 42.67 years. Of them, 60%, 22.7%, 14.7%, and 1.3% had pheochromocytoma, Cushing's syndrome, Conn's syndrome, and insulinoma, respectively. Malignancy was associated with greater tumor size (p<0.000) and mitotic rate (p=0.046), and the presence of necrosis (p=0.001), and capsular and vascular invasion (p<0.000). Clinical follow-up visits of 45 patients indicated 84.4% showed a complete response to the treatment (surgical ± medical). Treatment response was significantly influenced by approach (laparoscopic favored; p=0.001), surgery duration (<150 minutes better; p=0.017), mass pathology (adenoma favored; p=0.034), and capsular invasion (absence better; p=0.012).

**Conclusion:**

Adrenalectomy outcomes were significantly affected by surgical approach and tumor pathology, notably capsular invasion. Larger studies are needed to determine the predictive values of clinical and pathologic variables in adrenalectomy for adrenal tumors.

## Introduction

Adrenal masses represent a prevalent occurrence among endocrine tumors, with a prevalence of approximately 4-7% in individuals aged above 40 and reaching 5-10% in those aged above 70 (1, 2). These masses can be categorized into two groups based on their anatomic origins: cortical tumors, encompassing adrenocortical adenoma, cortical hyperplasia, Adrenocortical Carcinoma (ACC), and myelolipoma, and medullary tumors, including Pheochromocytoma (PHEO) (3-5). Recent research findings indicate that more than 90% of adrenal tumors exhibit benign pathological features, with adenomas being the most frequently encountered subtype (1, 6). Malignant tumors account for 5-8.6% of adrenal cases, with ACC being the most commonly diagnosed pathology (1, 7). Additionally, other identified malignant pathologies included adrenal metastasis, neuroblastoma, and lymphoma (1, 8-10). 

Moreover, previous studies demonstrated that most of the adrenal masses are non-functional, while 4.1% to 30.1% of adrenal tumors are reported to lead to hormone excess (functional tumors) (1, 11-13). The most frequently produced hormones by functional adrenal tumors include catecholamines, cortisol, and aldosterone, resulting in PHEO, Cushing’s syndrome, and primary aldosteronism (Conn’s syndrome), respectively (14, 15).

Adrenalectomy, a surgical procedure involving the resection of the adrenal gland and its associated mass, represents one of the most frequently performed endocrine surgeries, ranking third in prevalence after thyroid and parathyroid surgeries (16). The indications for adrenalectomy encompass three main categories: (a) functional adrenal tumors, regardless of their size, (b) suspicion of malignancy or the presence of malignant tumors such as ACC, malignant PHEO, and metastatic tumors, and (c) non-functional tumors with a malignancy risk, typically referring to tumors larger than 4 cm in diameter (17-20). The surgical approaches for adrenalectomy primarily involve open or laparoscopic techniques (21). Laparoscopic adrenalectomy has emerged as the gold-standard treatment for non-malignant adrenal tumors due to its associated benefits, including reduced surgery time, decreased blood loss, lower operation-related complications, and improved clinical outcomes for patients (18, 22, 23). Conversely, open adrenalectomy remains the preferred treatment for malignant adrenal masses, as it is associated with better oncological outcomes in these cases (24, 25).

Despite being one of the most frequently performed endocrine surgeries, the outcomes of adrenalectomy and their clinical and pathological determinants remain insufficiently characterized. This study aims to evaluate both short- and long-term outcomes following adrenalectomy through a comprehensive review of clinical records and pathological specimens. By correlating surgical outcomes with detailed demographic, clinical, and pathological variables, we seek to identify key prognostic factors that may guide clinical decision-making and improve patient management. The study will specifically analyze outcome differences between tumor subtypes, functional status, and surgical approaches to provide evidence-based recommendations for this common yet understudied procedure.

## Materials and Methods

### Study Design and Setting

The study employed a retrospective cohort design with consecutive sampling, encompassing all eligible patients who underwent adrenalectomy at the esteemed endocrine surgery referral center, Shariati Hospital, affiliated with Tehran University of Medical Sciences, Tehran, Iran, within the timeframe spanning from March 20, 2016, to March 20, 2021. Identification of eligible participants was achieved by meticulously examining both physical paper records and electronic databases. Subsequently, relevant information about the patients was extracted from the gathered data. To further supplement the data collection process, medical interviews were conducted with patients via telephone, allowing for an evaluation of their long-term treatment outcomes. This study strictly adhered to the guidelines and recommendations outlined in the Reporting of Studies Conducted using Observational Routinely-collected Health Data (RECORD) (26) and Strengthening the Reporting of Observational Studies in Epidemiology (STROBE) (27) checklists for the comprehensive reporting of observational studies. 

### Data Collection

For the data extraction process, a predetermined checklist was designed by an experienced epidemiologist at our center to comprehensively assess and gather demographic, clinical, and pathological information from each participant. By reviewing the medical records, we extracted the following data from each patient: (a) Demographic information: age at admission, biological sex, medical history, and family history of related disorders, (b) Clinical information: clinical manifestations, laterality of adrenal tumors, and secretory function of adrenal tumors, presence or absence of extra-adrenal involvements, preoperative Systolic Blood Pressure (SBP) and Diastolic Blood Pressure (DBP), adrenalectomy approach, presence or absence of extra-adrenal surgery, surgery duration, postoperative complications, and postoperative SBP and DBP, and (c) Pathologic information: adrenal tumor size (maximum diameter), pathology of the adrenal mass (including PHEO, adenoma, hyperplasia, and ACC), margin evaluation, mitotic rate, and presence or absence of necrosis, capsular and vascular invasion, calcification, and reactive lymph nodes. Moreover, during the clinical follow-up, outcome data were collected via structured telephone interviews conducted using a standardized checklist designed by an epidemiologist. This telephone-based follow-up protocol systematically collected the following data from each patient: symptom remission state (categorized as complete remission, partial remission, not changed, worsened, or dead), presence/absence of re-adrenalectomy, usage of relevant medication, follow-up SBP, and DBP. 

### Statistical Analysis

For statistical analysis, we utilized IBM^®^ SPSS^®^ Statistics software, version 25. The categorical data were presented as numbers (percentages) and continuous as mean ± standard deviation (range). To examine the associations between qualitative variables, the Chi-Square test was employed, with a predetermined level of statistical significance set at p<0.05. Additionally, we computed Cramer’s Phi (for 2*2 tables) and V (for non-2*2 tables) to estimate the effect size of the measured variables that attained statistical significance, with value=0.1, 0.3, and 0.5 interpreted as small, medium, and large effect sizes, respectively. Moreover, to evaluate the effects of both qualitative and quantitative variables on each other, we conducted Independent T-Tests and Analysis of Variance (ANOVA). Once again, the statistically significant level was set at p<0.05, and Cohen's d was computed to gauge the effect size of the measured variables that achieved statistical significance, with value=0.2, 0.5, and 0.8, which were interpreted as small, medium, and large effect sizes.

### Ethical Considerations

The research protocol received approval from the Ethical Committee and Institutional Review Board (IRB) of Tehran University of Medical Sciences, with a reference code of IR.TUMS.MEDICINE.REC.1400.1065. Also, this study strictly adhered to the ethical principles outlined in the Declaration of Helsinki (28). Before conducting the clinical interviews, verbal consent was obtained from each patient, emphasizing their voluntary participation and understanding of the study's purpose.

## Results

### Participants

**Fig. 1:**
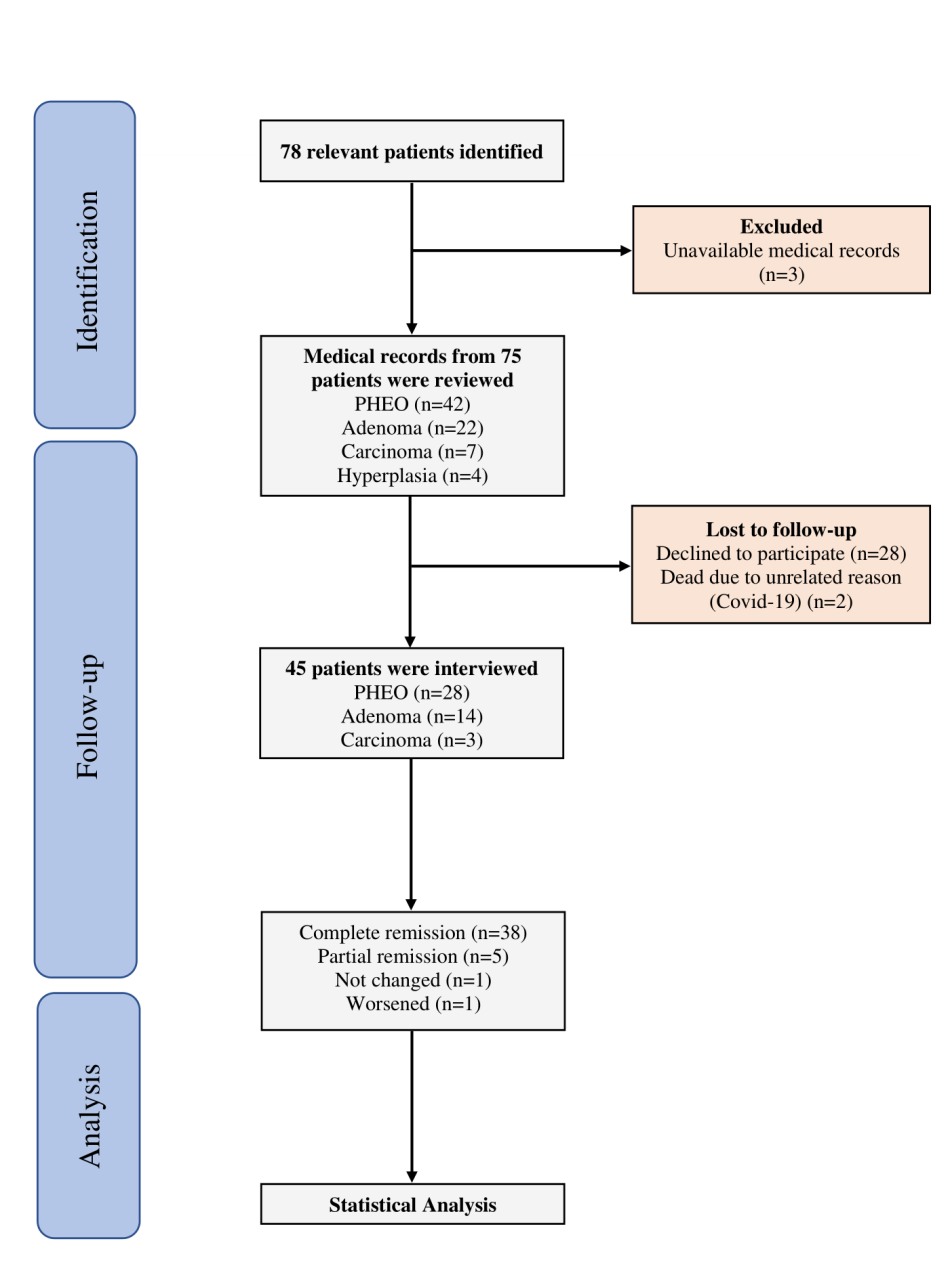
Flow diagram of the process of the selection and review of the patients’ data.

Between March 20, 2016, and March 20, 2021, 78 patients underwent adrenalectomy at our center. Due to unavailable medical records for 3 patients, 75 were included in this study (Fig. 1). The mean age was 42.67±13.45 years, with 55 females (73.3%) and 20 males (26.7%) (Table 1).

Hypertension (40%), thyroid gland disorders (33.3%), and diabetes mellitus (29.3%) were the most common medical conditions. Multiple Endocrine Neoplasia (MEN) disorders were reported in 14.6% of patients (MEN-I: 1.3%, MEN-II: 13.3%), secondary malignancies in 9.3%, hypopituitarism in 4%, neurofibromatosis in 2.7%, and Von Hippel-Lindau (VHL) in 1.3% (Table 1). Family history data were missing for 29 patients; among the rest, 11 had a positive family history of adrenal or thyroid pathologies.

**Fig. 2:**
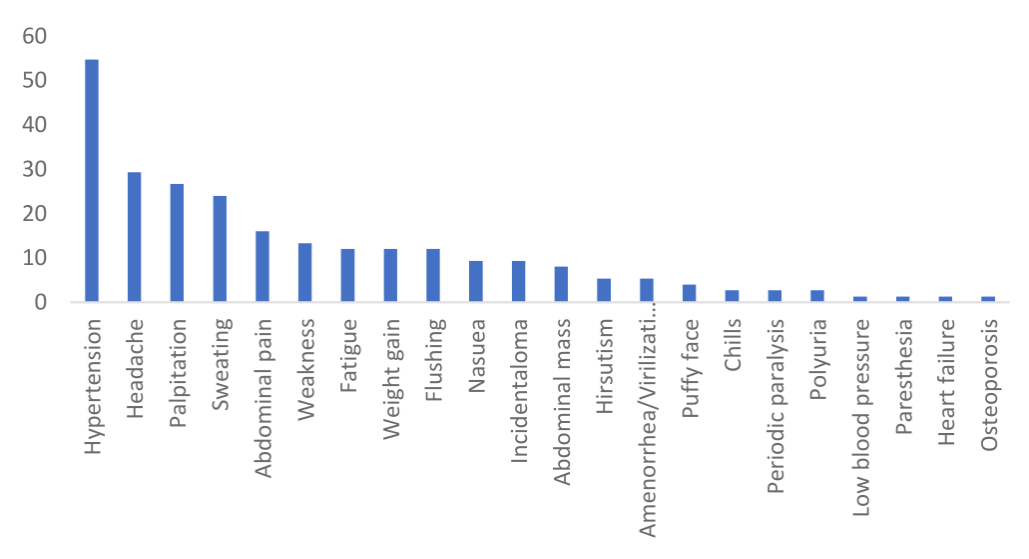
The prevalence of clinical presentations of adrenal masses across the study population.

Adrenal masses were incidentally found in 9.3% of cases (Incidentaloma). The most common symptoms were hypertension (54.7%), headache (29.3%), palpitation (26.7%), and sweating (24.0%). Regarding the secretory function of adrenal masses, 45 patients (60%) had PHEO, 17 (22.7%) had Cushing’s Syndrome, 11 (14.7%) had Conn’s Syndrome, 1 (1.3%) had insulinoma, and 1 (1.3%) had a non-functional mass (Fig. 2). Additionally, 8 patients (10.7%) had extra-adrenal masses in the pancreas, kidney, liver, inferior vena cava (IVC), gallbladder, renal artery, and thyroid. The preoperative mean systolic and diastolic blood pressures were 140.99±27.08 and 87.09±17.56 mmHg, respectively (Table 1).

**Table 1:** Characteristics of the included patients.

Variables	Values
Mean age at admission, y	42.67±13.45 (15-73)
Age groups	< 55 (n=56) ≥ 55 (n=19)
Biological sex	Female (n=55)
	Male (n=20)
Past medical history	Hypertension (n=30)
	Thyroid disorder (n=25)
	Diabetes mellitus (n=22)
	MEN-II (n=10)
	Secondary malignancy (n=7)
	Hypopituitarism (n=3)
	Neurofibromatosis (n=2)
	MEN-I (n=1)
	VHL (n=1)
Adrenal mass lateralization	Right (n=33)
	Left (n=34)
	Bilateral (n=8)
Extra-adrenal mass	Present (n=8)
	Absent (n=67)
Secretory function of adrenal masses	PHEO (n=45)
	Cushing (n=17)
	Aldosteronism (n=11)
	Insulinoma (n=1)
	Non-functional (n=1)
Mean preoperative SBP, mmHg	140.99±27.08 (80-230)
Mean preoperative DBP, mmHg	87.09±17.56 (40-160)

MEN-I: multiple endocrine neoplasia type I, MEN-II: multiple endocrine neoplasia syndrome type II, PHEO: pheochromocytoma, VHL: von Hippel-Lindau, y: years.

### Adrenalectomy Data

A total of 52 (69.3%) patients underwent laparoscopic adrenalectomy, while 23 (30.7%) underwent open adrenalectomy. Also, 5 (6.7%) patients underwent an additional extra-adrenal mass resection surgery, which included distal pancreatectomy, partial nephrectomy, partial colectomy, splenectomy, IVC tumor resection, and ex vivo resection of liver and IVC mass and autotransplant of liver and IVC shunt. The mean duration of the surgery was 154.33±82.11 minutes, with 40 (53.3%) surgeries lasting for 150 minutes or more. Moreover, 6 (8%) patients experienced postoperative complications, which included pleural effusion, atelectasis, pulmonary thromboembolism, and COVID-19 infection (leading to death in one patient. Also, the postoperative mean SBP and DBP of the patients were 115.49±12.09 and 71.81±9.50 mmHg (Table 2).

**Table 2:** Summary of adrenalectomy-related and pathologic data of the included patients.

Variables	Values
Surgery approach	Open (n=23)
	Laparoscopic (n=52)
Extra-adrenal surgery	Performed (n=5)
	Not performed (n=70)
Mean surgery duration, m	154.33 ± 82.11 (30-570)
Postoperative complications	Present (n=6)
	Absent (n=69)
Mean post-operative SBP, mmHg	115.49±12.09 (83-145)
Mean post-operative DBP, mmHg	71.81±9.50 (40-95)
Adrenal mass pathology	PHEO (n=42)
	Adenoma (n=22)
	Carcinoma (n=7)
	Hyperplasia (n=4)
Mean adrenal mass size, cm	6.62±5.05 (1-25)
Mean mitotic rate	2.24±2.38 (0-10)
Tumor margin	Well-defined (n=34)
	Irregular (n=11)
	Missing (n=30)
Necrosis	Present (n=11)
	Absent (n=38)
	Missing (n=26)
Capsular invasion	Present (n=10)
	Absent (n=37)
	Missing (n=28)
Vascular invasion	Present (n=7)
	Absent (n=41)
	Missing (n=27)
Calcification	Present (n=1)
	Absent (n=23)
	Missing (n=51)
Reactive lymph nodes	Present (n=2)
	Absent (n=24)
	Missing (n=49)

cm: centimeters, DBP: diastolic blood pressure, m: minutes, PHEO: pheochromocytoma, SBP: systolic blood pressure.

### Pathological Data

**Fig. 3:**
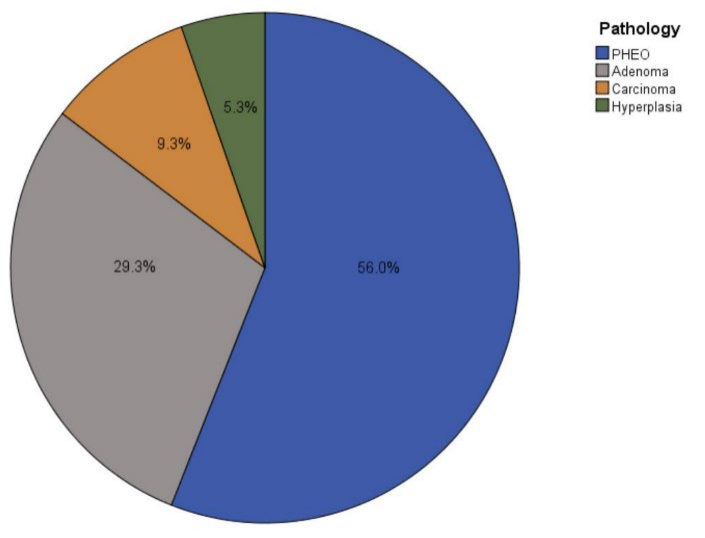
Pie chart illustrating the percentages of each adrenal pathology among the reviewed patients.

The mean size of the resected adrenal masses was 6.62±5.05 cm. Pathology reports indicated that 42 patients (56%) had medullary paraganglioma (PHEO), 22 (29.3%) had adrenocortical adenoma, 7 (9.3%) had ACC, and 4 (5.3%) had cortical hyperplasia (Table 2, Fig. 3).

**Fig. 4:**
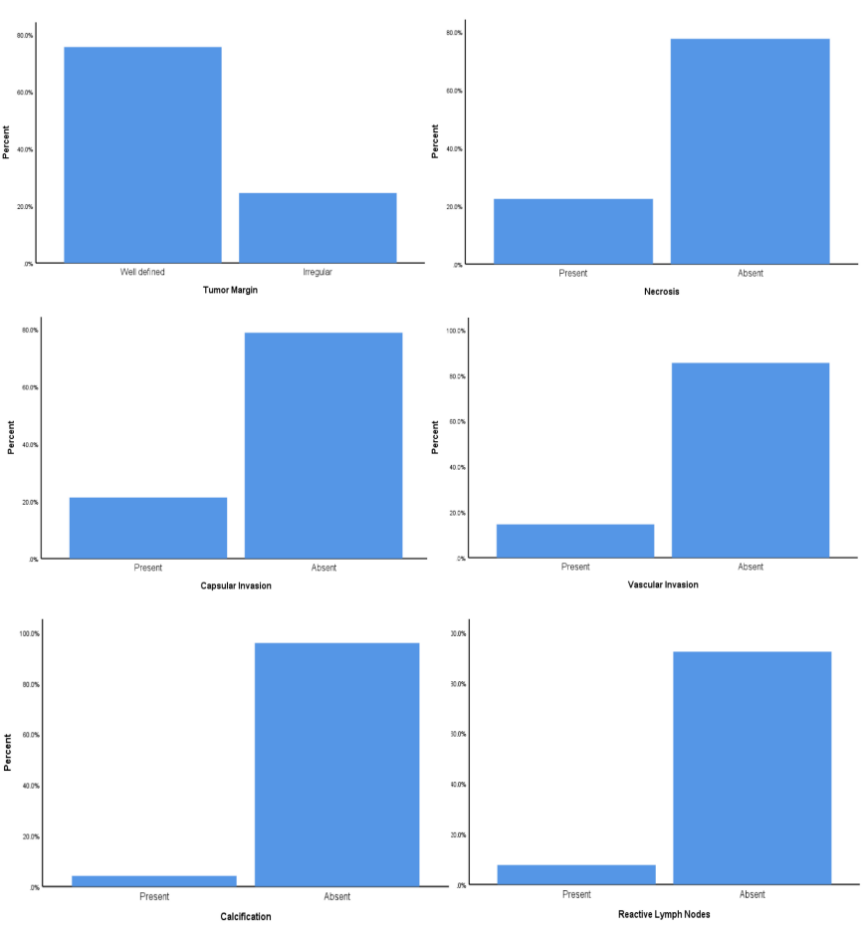
Bar charts represent the prevalence of different histopathological features in the analyzed cohort (the missing data were excluded).

 Histopathologic measures of the adrenal masses were as follows (Fig. 4): (1) 34 out of 45 (75.6%) had well-defined margins, while 11 out of 45 (24.4%) had irregular margins; (2) the mean mitotic rate was 2.24±2.38 mitotic figures per 10 high-power fields (reported from 45 patients); (4) necrosis, capsular invasion, vascular invasion, calcification, and reactive lymph nodes were present in 11 out of 49 (22.4%), 10 out of 47 (21.3%), 7 out of 48 (14.6%), 1 out of 24 (4.2%), and 2 out of 26 (7.7%) of the adrenal masses, respectively. It is noteworthy that during the record review, a substantial portion of patients lacked complete pathology data. These cases were documented as missing values and consequently excluded from relevant analyses (Table 2).

### Follow-up Data

Of the 75 patients initially enrolled in the study, follow-up data for 28 were missing due to unresponsiveness. Additionally, 2 patients had died due to COVID-19, and their information was excluded from further analysis, leaving 45 participants. The mean follow-up interval for these patients was 1555.98±544.90 days from their first operation. Among these, 38 patients (84.4%) experienced complete remission, 5 (11.1%) had partial remission, 1 (2.2%) had no remission with disease recurrence and refractory to treatments, and 1 (2.2%) had worsened symptoms. Overall, 38 patients (84.4%) showed a complete response to treatment (CRT), while 7 (15.6%) showed an incomplete response (NCRT). Furthermore, 2 patients (4.4%) underwent a second adrenalectomy due to a lack of response to the initial surgery and disease recurrence (Table 3).

Lastly, 29/45 (64.4%) reported the regular use of medications after the surgery, while 16/45 (35.6%) did not mention the use of any related medications. Additionally, according to the patients’ reports in the follow-up calls, the mean SBP and DBP of the patients in the long-term postoperative period were 117.90±9.25 and 79.80±8.11 mmHg, respectively (Table 3).

**Table 3:** Summary of adrenalectomy-related and pathologic data of the included patients.

Variables	Values
Mean follow-up interval, d	1555.98±544.90 (828-2521)
Long-term treatment response status	Complete (n=38)
	Partial (n=5)
	Not changed (n=1)
	Worsened (n=1)
	Missing (n=30)
Second adrenalectomy	Performed (n=2)
	Not performed (n=43)
	Missing (n=30)
Related medication usage	Positive (n=29)
	Negative (n=16)
	Missing (n=30)
Mean follow-up SBP, mmHg	117.90±9.25 (100-130)
Mean follow-up DBP, mmHg	79.80±8.11 (60-100)

cm: centimeters, d: days, DBP: diastolic blood pressure, SBP: systolic blood pressure.

### Statistical Analysis

#### Symptoms at Presentation

Hypertension (p=0.009), sweating (p=0.007), and nausea (p=0.047) were found more frequently among patients aged≥55 years. Abdominal mass and headache were also more frequent in this aging group, but failed to reach a statistical significance level (p>0.05). On the other hand, weakness (p=0.002) and fatigue (p=0.001) were more common in patients aged<55 years. Also, weight gain, puffy face, abdominal pain, and palpitation were more frequent among the younger group of patients but failed to reach a statistical significance level (p>0.05).

### Pathologic Features

The mean size of tumors with ACC pathology was 17.50±6.72 cm, while those with PHEO, adenoma, and hyperplasia measured 6.79±4.37 cm, 3.94±2.30 cm, and 6.00±1.35 cm, respectively. There was a significant difference in tumor size based on pathology (p<0.000). Additionally, pathology significantly affected the presence of necrosis (p=0.001), capsular invasion (p<0.000), and vascular invasion (p<0.000), with ACC showing the highest rates. Margin characteristics also differed significantly among pathologic groups (p=0.028), with ACC exhibiting the highest rates of irregular margins. The mean mitotic rate was significantly higher in patients with ACC (7.33±2.51 mitotic figures per 10 high-power fields) compared to those with adenoma (2.56±3.32 mitotic figures per 10 high-power fields) and PHEO (1.7±1.18 mitotic figures per 10 high-power fields) (p<0.000) (Table 4).

**Table 4:** Summary of adrenalectomy-related and pathologic data of the included patients.

Variables	Pathologic groups				Group effect	Effect size
	ACC	PHEO	Adenoma	Hyperplasia		
Mean size, cm	17.50±6.72	6.79±4.37	3.94±2.30	6.00±1.35	p<0.000	Medium to large (d=0.694)
Mean mitotic rate	7.33±2.51	1.70±1.85	2.56±3.32	NA	p=0.046	Medium to large (d=0.606)
Atypic cells	Present (n=2) Absent (n=0)	Present (n=2) Absent (n=17)	Present (n=2) Absent (n=4)	NA	p=0.011	Very large (Cramer’s V=0.575)
Necrosis	Present (n=4) Absent (n=0)	Present (n=6) Absent (n=31)	Present (n=1) Absent (n=7)	NA	p=0.001	Very large (Cramer’s V=0.555)
Capsular invasion	Present (n=5) Absent (n=0)	Present (n=4) Absent (n=31)	Present (n=1) Absent (n=6)	NA	p<0.000	Very large (Cramer’s V=0.664)
Vascular invasion	Present (n=5) Absent (n=0)	Present (n=2) Absent (n=34)	Present (n=0) Absent (n=7)	NA	p<0.000	Very large (Cramer’s V=0.827)
Calcification	NA	Present (n=0) Absent (n=19)	Present (n=1) Absent (n=4)	NA	NS	NA
Reactive lymph nodes	NA	Present (n=1) Absent (n=19)	Present (n=1) Absent (n=5)	NA	NS	NA

cm: centimeters, m: minutes, NA: not available, NS: non-significant, PHEO: pheochromocytoma

### Surgery Technique

The mean sizes of the adrenal mass in the patients that underwent laparoscopic and open adrenalectomy were 5.28±3.43 and 9.75±6.77 cm, respectively, with a significant between-group difference (p=0.006). Also, 49/58 of the patients with masses<10cm and 3/13 patients with masses≥10cm underwent laparoscopic surgery (p<0.000). 

Moreover, the duration of the surgery was significantly higher in the open adrenalectomy group (p<0.000). Also, the Chi-Square analysis revealed a higher prevalence of operations with ≥150 minutes duration in the open surgery group (p<0.000). Furthermore, there was no significant difference between the secretory function of the adrenal tumors and the deployed adrenalectomy approach (p>0.05).

However, the effect of the surgical approach on the occurrence of post-operation complications did not reach a statistical significance level (p=0.110). Also, no significant effect was seen for the duration of the surgery on the occurrence of post-operation complications (p=0.156).

### Treatment Response

Table 5 presents the results of statistical analyses assessing the effects of various features on patient response to adrenalectomy. The primary analysis showed significant differences in treatment response (CRT vs. NCRT) among different pathologic adrenal masses (PHEO, adrenocortical adenoma, and ACC), with ACC showing the worst and adrenocortical adenoma showing the best outcomes (p=0.034).

Additional Chi-Square tests evaluated the impact of demographic, clinical, and pathological variables on treatment response across the whole sample and among patients with specific pathologies (PHEO and adrenocortical adenoma). The ACC group was excluded from further analysis due to the limited number of participants. The results indicated that treatment response in the whole sample was significantly influenced by the type of adrenalectomy (p=0.001), timing of the adrenalectomy (p=0.017), size of the adrenal masses (p=0.014), and presence of capsular invasion (p=0.012).

However, other demographic, clinical, and pathological variables did not significantly affect treatment response (p>0.05). In the PHEO and adrenocortical adenoma groups, significant factors affecting treatment response included the adrenalectomy approach (p=0.007), timing of the adrenalectomy (p=0.044), and presence of capsular invasion (p=0.009).

To assess the effects of quantitative variables (age at admission, surgery duration, tumor size, and mitotic rate of the adrenal mass) on treatment outcomes, an Independent T-Test analysis was performed on the whole sample (Table 5) and the two major pathologic groups (PHEO and adrenocortical adenoma).

For the whole sample, treatment response was significantly affected by surgery duration (p=0.014) and tumor size (p=0.005). However, age at admission and mitotic rate did not significantly impact treatment response (p>0.05). Further analysis within the PHEO and adrenocortical adenoma groups revealed no significant effects of age at admission, surgery duration, tumor size, or mitotic rate on treatment response (p>0.05).

Additionally, the effect of the adrenalectomy approach on postoperative and follow-up blood pressure was examined in the whole sample and the PHEO group. Patients who underwent laparoscopic adrenalectomy had lower mean postoperative and follow-up SBP and DBP. However, only the differences in postoperative DBP reached statistical significance in both the whole sample (p=0.049) and the PHEO group (p=0.030).

**Table 5:** Significant differences in different variables between the CRT and NCRT patients of the whole sample.

Variable	P-value	Effect size^a^
Adrenal mass laterality: Right, Left, and Bilateral	NS^b^	NA
Age (continuous)	NS^c^	NA
Aging groups: ≥55 and <55 years	NS^b^	NA
Biological sex: Male and Female	NS^b^	NA
Adrenalectomy approach: Open and Laparoscopic	0.001^b^	Medium to large
Pathological groups: PHEO, Adenoma, and Carcinoma	0.034^b^	Medium to large
Surgery duration (continuous)	0.014^c^	Very small
Surgery duration groups: ≥150 and <150 minutes	0.017^b^	Medium to large
Adrenal mass size (continuous)	0.005^c^	Very small
Adrenal mass size groups: ≥10 and <10 centimeters	0.014^b^	Medium to large
Presence/Absence of capsular invasion	0.012^b^	Medium to large
Other pathologic features; e.g., mitotic rate, margin, vascular invasion, etc.	NS^b^	NA

CRT: complete response to the treatment, NA: not assessed, NCRT: incomplete response to the treatment, NS: statistically non-significant, PHEO: pheochromocytoma.

a. Effect sizes were estimated as follows: (1) for the Chi-Square tests, the effect sizes were estimated by calculating the Cramer’s Phi (for 2⁕2 tables) and Cramer’s V values (for non- 2⁕2 tables), which value=0.1, 0.3, and 0.5 were interpreted as small, medium, and large effect sizes, respectively; and (2) for the independent T-Tests, the effect sized were estimated through calculating the Cohen’s d values, which value=0.2, 0.5, and 0.8 were interpreted as small, medium, and large effect sizes.

b. Chi-square test.

c. Independent T-Test.

## Discussion

Based on our findings, the clinical presentation of the adrenal tumors is different between the different aging groups, with a higher prevalence of hypertension, nausea, and sweating in older patients, and weakness and fatigue in younger patients. Also, as expected, patients with ACC had the worst long-term outcomes compared to the other adrenal tumor pathologies. Moreover, the long-term treatment outcome was better in patients who underwent laparoscopic adrenalectomy, had shorter surgery duration, had smaller adrenal tumors, and did not have capsular invasion. 

Furthermore, our study findings have elucidated significant differences between malignant and benign adrenal tumors. Notably, malignant tumors exhibited larger diameters, increased mitotic activities, and a higher occurrence of necrosis, as well as capsular and vascular invasion. These observations corroborate previous research, which also reported that the presence of tumor necrosis and higher mitotic rates is indicative of a heightened risk of malignancy in adrenal tumors (29). Also, our investigation unveiled a significant association between capsular invasion and unfavorable long-term outcomes in patients who underwent adrenalectomy, aligning with the conclusions drawn by Picard et al. (30). However, it is worth noting that Picard et al. (30) identified tumor necrosis, vascular invasion, and mitotic rate as significant predictors of treatment outcomes, in contrast to our analysis, which did not find them to be statistically significant predictors. These findings underscore the importance of considering capsular invasion as a crucial pathologic biomarker for predicting treatment response. Nevertheless, there remains a need for further research to fully comprehend the applicability of other pathologic features in this domain. 

Moreover, it is noteworthy that the average size of resected adrenal tumors in patients who underwent open adrenalectomy was found to be significantly greater than those who opted for the laparoscopic approach. Interestingly, among the patients diagnosed with PHEO and having adrenal tumors larger than 10 cm in diameter, three individuals underwent laparoscopic adrenalectomy. Although these PHEO patients declined to participate in the long-term follow-up process, their post-operation SBP ranged from 114 to 120 mmHg, and DBP ranged from 65 to 80 mmHg, both showing significant reductions. Notably, these blood pressure changes have previously been identified as significant predictors of long-term treatment outcomes in patients. While current guidelines recommend open adrenalectomy for PHEOs >8 cm (25), these findings suggest laparoscopic resection may remain viable for select large PHEOs when performed by experienced surgeons. Surgical approach decisions should therefore integrate multiple factors, including: (1) tumor characteristics (size, localization, vascular involvement), (2) surgical team expertise, and (3) patient comorbidities, rather than relying solely on size thresholds. This supports individualized, patient-centered decision-making for large PHEOs.

In accordance, Gan et al. (31) have recently demonstrated favorable outcomes for the laparoscopic approach in patients with PHEO, in comparison to open adrenalectomy. Also, a recent study revealed comparable clinical outcomes in PHEO patients with tumors larger than 10 cm who underwent open or laparoscopic adrenalectomy (32). Therefore, larger studies employing comparative designs are warranted to thoroughly investigate the feasibility and effectiveness of the laparoscopic approach for the management of larger adrenal tumors, specifically PHEO. Such research endeavors would contribute to a more evidence-based approach to the treatment decision-making process in patients with substantial adrenal tumors.

Our study has several significant limitations. Firstly, the included population is relatively limited, and we encountered a considerable amount of missing data due to incomplete pathologic reports and patients' unwillingness to participate in the clinical follow-up interview. Moreover, our investigation primarily focused on adrenalectomy outcomes in patients with functional tumors, overlooking a substantial percentage of adrenalectomies performed on patients with incidentalomas. Assessing the treatment outcomes in these patients is essential for a comprehensive understanding of the subject. Additionally, we did not evaluate the association between different radiologic features of adrenal tumors and adrenalectomy outcomes, which is an important aspect to consider. Given these limitations, further prospective studies with larger sample sizes and more comprehensive radiologic and histopathological data collection are warranted.

## Conclusion

Our findings demonstrate that long-term adrenalectomy outcomes are significantly influenced by tumor characteristics (size, pathologic subtype, and capsular invasion) and surgical factors (approach and duration). These results highlight three key clinical considerations: (1) comprehensive preoperative assessment using advanced imaging and biochemical profiling is essential for surgical planning; (2) laparoscopic adrenalectomy should be strongly considered for smaller (<6 cm), non-invasive tumors given its documented advantages in recovery and outcomes; and (3) patients with capsular invasion or larger tumors (>6 cm) require intensified postoperative surveillance due to their higher complication risk. By incorporating these strategies into clinical decision-making, surgeons can better predict and optimize long-term treatment effectiveness for adrenal tumor patients.

## Data Availability

All data generated during the study are included in this article. Further enquiries can be directed to the corresponding author.
